# Awareness and experiences of cosmetic treatment providers with body dysmorphic disorder in Saudi Arabia

**DOI:** 10.7717/peerj.8959

**Published:** 2020-04-21

**Authors:** Abdullah E. Kattan, Nujaim H. Alnujaim, Omar Barasain, Theo K. Bouman, Reema AlHammad, Berend Van der Lei

**Affiliations:** 1 Division of Plastic Surgery, Department of Surgery, College of Medicine, King Saud University, Riyadh, Saudi Arabia; 2 College of Medicine, King Khalid University Hospital, King Saud University, Riyadh, Saudi Arabia; 3 Department of Clinical Psychology, University of Groningen, Groningen, The Netherlands; 4 Department of Plastic, Reconstructive, Aesthetic, and Hand Surgery, University Medical Center Groningen, Groningen, The Netherlands

**Keywords:** Body dysmorphic disorder, Awareness of body dysmorphic disorder, Cosmetic treatment providers

## Abstract

Body Dysmorphic Disorder (BDD) is defined as a constant obsession with one’s external appearance and flaws, and it falls under the criteria of neuropsychiatric disorders. Individuals suffering from this disorder may seek unnecessary cosmetic procedures from cosmetic treatment providers such as dermatologists or plastic surgeons. Cosmetic treatments have become readily available, which has led to an influx of undiagnosed BDD patients electing to undergo such treatments. Therefore, physicians should have the clinical knowledge about BDD to diagnose and manage these cases to avoid psychological and physical harm to these patients. However, there were no studies conducted in our region to assess the awareness of BDD among physicians who provide cosmetic treatments with regards to their attitude toward such cases and how they would manage it. This study aims to assess the awareness of Body Dysmorphic Disorder among Saudi physicians who provide cosmetic treatments. We conducted an observational cross-sectional study among physicians practicing in hospitals and cosmetic clinics in Riyadh and Jeddah city (Saudi Arabia), who perform cosmetic procedures, namely dermatologists, plastic surgeons, and otorhinolaryngologists. A paper-based questionnaire consisting of multiple-choice questions was distributed among them. The total number of participants was 155 physicians: 113 (72.9%) males and 42 (27.1%) females. Eighty-two (52.9%) participants reported that they have been familiar with the diagnostic criteria of BDD for a long time and ninety-nine (63.8%) reported being familiar with the clinical picture of BDD. Sixty-three (40.6%) participants estimated the prevalence of BDD cases seen in cosmetic practice to range from 1%-5%, and most agreed on an equal prevalence of BDD among female and male patients. Half of the participants (*n* = 76) (49%) reported that they sometimes share knowledge about BDD with patients whom they suspect to suffer from this condition. In conclusion, cosmetic treatment providers in Saudi Arabia are aware of BDD, but we have identified a discrepancy between the self-reported participant knowledge of diagnostic criteria and their ability to accurately estimate the prevalence of BDD cases seen in clinical practice.

## Introduction

Body Dysmorphic Disorder (BDD) is a neuropsychiatric disorder characterized by an irrational dissatisfaction with one’s external appearance along with a constant obsession over perceived physical flaws that might seem like a major defect to the patient while appearing unnoticeable or minor to others. There are multiple symptoms and behavioral tendencies, all of which are related to repetitive preoccupation with physical appearance, which include: thinking about the perceived physical flaws, spending long hours of the day grooming, and constantly comparing one’s appearance with others (American Psychiatric Association, 1994).

Screening tools such as the Body Dysmorphic Disorder Questionnaire *(BDDQ)* (Phillips, 2005), and diagnostic tools such as the *DSM-5* (American Psychiatric Association, 2013), were indeed useful and have since been implemented and widely used to estimate the prevalence of BDD in specific patients and the general population. A recent systematic review of BDD in aesthetic plastic surgery patients in the United States showed a prevalence of 13% (Sweis et al., 2017), which is higher than the reported prevalence of 2.4% in the general US population (Koran et al., 2008). Moreover, a literature review has shown a BDD prevalence of 8.5% to 21% among dermatology patients (Koran et al., 2008). While seeking cosmetic treatment is a symptom of BDD, a study conducted in 2002 has shown that the perceived prevalence of BDD in plastic surgery patients by members of the American Society for Aesthetic Plastic Surgery *(ASAPS)* was underestimated and believed to be 2.3% (Sarwer, 2002).

Cosmetic surgery and other aesthetic treatments are rapidly growing fields in Saudi Arabia. Although there is a marked increase in the popularity of cosmetic treatments and surgeries in Saudi Arabia, there is still a lack of studies examining the interaction of cosmetic physicians and patients suffering from BDD. Currently, only a few studies are exploring the topic of Body Dysmorphic Disorder in Saudi Arabia. One study included (453) Saudi patients seeking facial plastic surgery (Al Shuhayb, 2019), and the estimated prevalence was 14.19%. Two more studies were conducted on patients attending dermatology clinics in Riyadh city (AlShahwan, 2020), and Qassim region (Alonazi et al., 2017). BDD prevalence was found to be 14.1% and 18.6%, respectively. The Body Dysmorphic Disorder Questionnaire *(BDDQ)* was used as a screening tool in the previously mentioned studies (Alonazi et al., 2017; AlShahwan, 2020; Al Shuhayb, 2019). Another study (Al Arfaj et al., 2016), used a translated and validated Arabic version of the COPS *(Cosmetic Procedure Screening)* questionnaire to screen patients booked for cosmetic surgeries through an outpatient clinic and found that 6.6% of patients screened positive for BDD symptoms. The last study, by Shaffi Ahamed et al. (2016), on female medical students at King Saudi University, which used the BIDQ (*Body Image Disturbance Questionnaire)*, estimated a prevalence of 4.5%.

 The relatively high prevalence of BDD in cosmetic settings poses a challenge for healthcare professionals such as cosmetic surgeons. In a recent study in The Netherlands (Bouman, Mulkens & Van der Lei, 2017), it was found that many cosmetic professionals reported being aware of the clinical picture and diagnosis of BDD, but that they hardly identified any patients with these disorders. Also, only a minority of the professionals explored body image problems during their first interview with patients, thereby neglecting a potential psychological contra-indication for cosmetic procedures. Hence, this study aims to assess the awareness of Body Dysmorphic Disorder among physicians who provide cosmetic treatments in Saudi Arabia. Additionally, we sought to explore the physician’s attitude, and experiences with such patients.

## Materials and Methods

We conducted an observational cross-sectional study with convenience sampling, as we anticipated a low response rate as seen in previous studies on similar participants. Inclusion criteria were: Board-certified Plastic Surgeons, Dermatologists, and Otorhinolaryngologists who provide surgical and non-surgical cosmetic treatments and are practicing in hospitals and private clinics. The study was carried out in the city of Riyadh and Jeddah, Saudi Arabia from December 2017 to June 2018.

The sample size was calculated using a (94%) awareness of the diagnostic criteria of BDD reported in a previous study (Bouman, Mulkens & Van der Lei, 2017), at a (95%) confidence interval, and .05 degrees of freedom *n* = *Z*^2^_*α*_*p(1-p)/d* ^2^, *n* = (1.96)^2^(.94) × (.06)/(.05)^2^ = 87. The sample size obtained was increased in an attempt to enhance the precision of the results.

We have constructed a questionnaire that is primarily based on Bouman et al. study which assessed the awareness of the members of Dutch professional associations for aesthetic plastic surgery, dermatology, and cosmetic medicine of Body Dysmorphic Disorder (Bouman, Mulkens & Van der Lei, 2017), with minor modifications to fit the purpose of our study. Possible participants were located in various institutions in Riyadh and Jeddah city and paper-based questionnaires were handed to the relevant departments and collected later for analysis. The questionnaire consisted of close-ended questions that were divided into 4 sections. The first section inquired about demographic information and characteristics of the respondents. The second section inquired about familiarity with Body Dysmorphic Disorder. The third section inquired about the respondent’s attitudes towards BDD, and the fourth section focused on the way participants dealt with patients whom they have suspected to have been suffering from body dysmorphic disorder. The specific questions of each section are presented in Tables 1 to 4. Permission from the institutional review board of King Saud University was obtained before conducting the study (*Approval Number: E 17-2407*), and written informed consent was also obtained from each individual before participation.

**Table 1:** General charcteristics of the respondnts.

**Age**	Mean = 39.76 + −8.15
**Gender**	
**A. Male**	113(72.9%)
**B. Female**	42(27.1%)
**Speciality**	
**A. Plastic Surgeon**	56(36.1%)
**B. Dermatologist**	98(63.2%)
**C. Otorhinolaryngologist**	1(0.7%)
**Years of experience**	
**A. Less than 5**	56(36.1%)
**B. 5–10**	52(33.5%)
**C. 10–15**	34(21.9%)
**D. 15–20**	9(5.8%)
**E. More than 20**	4(2.6%)
**Number of new patients each year**	
**A. Less than 200**	57(36.8%)
**B. 200–350**	47(30.3%)
**C. 350–500**	34(21.9%)
**D. More than 500**	15(9.7%)
**Nationality**	
**A. Saudi**	125(80.6%)
**B. Non-Saudi**	30(19.4%)

The data was analyzed using *IBM SPSS version 22.0*. The association between participants’ answers and their familiarity with the diagnostic criteria of BDD (*Question 1*, Table 2), and the differences between different specialties was established using Chi^2^ statistics. A *p*-value < *0.05* was considered statically significant.

**Table 2:** Awareness of body dysmorphic disorder.

	**No. (%)**	**X2**	**df**	*p*
**1. Are you familiar with the diagnostic criteria of BDD?**		–	–	–
A. I’m seeing these criteria now for the first time	21 (13.5%)			
B. I’ve heard of these	29 (18.7%)			
C. I’m slightly familiar with these	23 (14.8%)			
D. I’ve been familiar with these for a long time	82 (52.9%)			
**2.Are you familiar with the Clinical picture of BDD?**				
A. Not familiar	19 (12.3%)			
B. Partly familiar	37 (23.9%)	120.356	9	.001
C. Reasonably familiar	47 (30.3%)			
D. Totally familiar	52 (33.5%)			
**3. How did you acquire knowledge on BDD?**				
A. General Literature	83 (53.5%)	19.351	3	.000
B. Specific Literature	23 (14.8%)	8.048	3	.045
C. Conferences or Lectures	22 (14.7%)	4.472	3	.215
D. Colleagues	16 (10.3%)	11.720	3	.008
E. Web Sites	16 (10.3%)	31.282	3	.000
**4. What do you estimate the prevalence of BDD is in cosmetic practice?**				
A.1%–5%	63 (40.6%)			
B.5%–10%	36 (23.2%)	37.389	12	.001
C.10%–15%	22 (14.2%)			
D.15%–20%	23 (14.8%)			
E. Don’t Know	11 (7.1%)			
**5. Male patients BDD**				
A.5%–10%	66 (42.6%)			
B.10%–20%	31 (20.0%)	24.808	9	.003
C. 20%–30%	11 (7.1%)			
D. Don’t know	46 (30.3%)			
**6. Female patients BDD**				
A.5%–10%	60 (38.7%)			
B.10%–20%	42 (21.7%)	26.126	9	.002
C. 20%–30%	34 (21.9%)			
D. Don’t know	19 (12.3%)			
**7. Did you encounter patients with BDD over the past year?**				
A.Probably	69 (44.5%)	19.152	6	.004
B.Certainly	63 (40.6%)			
C. No	23 (14.8%)			
**8. How many patients with BDD did you see last year?**				
A. None	23 (14.8%)			
B. 1–5	88 (56.8%)	20.330	9	.016
C. 5–10	15 (9.7%)			
D. More than 10	29 (18.7%)			

## Results

Table 1 represents general information about participants, total number of participants was 155, the majority were male, and dermatologist with a mean age of 40 years.

As shown in Table 2, more than half of the participated physicians reported to be familiar with the diagnostic criteria of Body Dysmorphic Disorder, and almost two-third claimed they were reasonably to totally familiar with what the clinical picture of BDD. Familiarity with the diagnostic criteria and the clinical picture of BDD was distributed equally between both plastic surgeons and dermatologists, with a *p*-value of <0.573 and <0.536, respectively. Participants mainly acquired their knowledge on BDD from general literature. The most commonly reported estimate of the prevalence of BDD in patients was 1–5%, with 70% of the participants seeing (0–5) BDD patients last year. It is statistically significant concerning the knowledge of the diagnostic criteria of BDD that 63 (40.6%) of participants claimed that they have certainly encountered patients with BDD over the past year.

Table 3 lists the participants’ opinions regarding 12 statements about aesthetic intervention for BDD. The highest mean score was for the eighth statement: “If I think an aesthetic procedure is unnecessary, I will tell the patient”. Most dermatologists (62%) strongly agreed to this statement but less than half of plastic surgeons (42%) strongly agreed to it, this difference was statistically significant *p* (<0.048). Lowest mean scores belonged to the fifth and ninth statements, stating “Patients have realistic expectations on the physical results of their aesthetic procedure”, and “Even if I think an aesthetic procedure is unnecessary, I would still carry out the procedure”, respectively. With regards to the third statement “ In patients with BDD, do you shift the topic from the technical aspects of the procedure to body image problems?” a strong statistically significant difference was found with a *p*-value (<0.008) with more plastic surgeons (44%) generally agreeing with this statement than dermatologists (34%).

**Table 3:** Attitudes toward body dysmorphic disorder and cosmetic surgery.

	**Response Categories**^a^					**Mean**	**SD**	**X2**	**df**	*p*
	1	2	3	4	5					
**1. I usually get a gut feeling that something is wrong when seeing patients with BDD**	7 (4.5%)	10 (6.5%)	46 (29.7%)	42 (27.1%)	50 (32.3%)	3.765	1.116	38.410	12	.001
**2. I find it challenging to deal with patients with BDD**	5 (3.2%)	7 (4.5%)	43 (27.7%)	36 (23.2%)	64 (41.3%)	3.961	1.081	21.744	12	0.04
**3. I find BDD a contraindication for an aesthetic procedure**	16 (10.5%)	14 (9.2%)	50 (32.7%)	27 (17.6%)	46 (30.1%)	3.487	1.304	22.602	12	.031
**4. Patients with BDD have realistic expectations on the impact of aesthetic procedure on their daily functioning**	65 (42.8%)	28 (18.4%)	35 (23.0%)	14 (9.2%)	10 (6.6%)	2.180	1.270	40.083	12	.001
**5. Patients with BDD have realistic expectations on the physical results of their aestheticprocedure**^b^	80 (51.6%)	19 (12.3%)	35 (22.6%)	13 (8.4%)	8 (5.2%)	2.020	1.248	31.198	12	.002
**6. Patients with BDD benefit equally from the aesthetic procedures as other patients**	47 (30.3%)	38 (24.5%)	54 (34.8%)	5 (3.6%)	11 (7.1%)	2.309	1.158	23.064	12	.027
**7. If a patient wants an aesthetic procedure, I will always carry it out**	58 (37.4%)	24 (15.5%)	49 (31.6%)	7 (4.5%)	17 (11.0%)	2.359	1.326	27.265	12	.007
8. If I think an aesthetic procedure isunnecessary, I will tell the patient**	12 (7.7%)	18 (11.6%)	18 (10.6%)	23 (14.8%)	84 (54.2%)	3.974	1.357	25.606	12	.012
**9. If I think an aesthetic procedure isunnecessary, I will still carry it out**	89 (57.4%)	23 (14.8%)	28 (18.1%)	1 (0.6%)	14 (9.0%)	1.876	1.263	20.490	9	.058
**10. If I think an aesthetic procedure isunnecessary, I will refer the patient to a colleague**	56 (36.1%)	19 (12.3%)	42 (27.1%)	14 (9.0%)	24 (15.5%)	2.549	1.455	7.231	12	.842
**11. Aesthetic procedures are a luxury article, but also patient care**	21 (13.5%)	13 (8.4%)	35 (22.6%)	31 (20.0%)	55 (35.5%)	3.569	1.399	40.465	12	.001
**12. Aesthetic procedures are basically a kind of “Psychotherapy/Psychosurgery”**	27 (17.4%)	5 (3.2%)	67 (43.2%)	27 (17.4%)	29 (18.7%)	3.170	1.286	18.671	12	.097

**Notes.**

a Level of agreement with the statement, where 1 represents “Do not agree at all”, 3 represents “Neutral”, and 5 represents “Totally Agree”.

b 5 and 8 ×Specialty *p* < .003 / *p* < .048 (statistically significant based on the different specialties).

Table 4 summarizes the encountered symptoms of BDD, how physicians interview patients with BDD, and how physicians choose to manage patients with BDD. The symptom most encountered was: Excessive concern with, or distress over, minor or nonexistent appearance flaws (62.6%) (*n* = 97). There is a statistically significant relation between the knowledge of the diagnostic criteria of BDD and whether or not the physician explores BDD during the initial interview with the patients (*p* = .006). Only 12.9% of the 155 surveyed participants (*n* = 20) reported exploring BDD during their initial interview with the patient. Most of the physicians who reported always exploring BDD during the initial interview indicated being familiar with the diagnostic criteria of BDD for a long time. Only five physicians (3.2%) reported they would carry out procedures in parallel with psychiatric care.

## Discussion

This study is the first to explore the cosmetic treatment providers’ awareness of Body Dysmorphic Disorder in Saudi Arabia. Similar to other international studies, most of our participants were aware of BDD (Bouman, Mulkens & Van der Lei, 2017; Sarwer, 2002; Sarwer et al., 2015; Szepietowski et al., 2008). Bouman, Mulkens & Van der Lei (2017) conducted a study on Dutch cosmetic professionals to assess the awareness of BDD. In comparison to the study by Bouman et al., a much higher percentage of our study participants (12.3%) reported being totally unfamiliar with the clinical picture of BDD, to only a single participant (0.6%) from the same study. Another study by Sarwer et al., (2015), on dermatologic surgeons, showed that only 8% of the participants were unaware of BDD.

Pertaining to the sources of knowledge on BDD, most participants reported acquiring their knowledge on BDD through general literature (*n* = 83, 53.5%), while most participants of the Dutch study acquired their knowledge on BDD through conferences and lectures, with general literature coming second (Bouman, Mulkens & Van der Lei, 2017).

Current study participants estimated the prevalence of BDD in cosmetic patients to be around 5%. This is similar to what was reported by Bouman, Mulkens & Van der Lei (2017). A study by Sarwer (2002), on American Society for Aesthetic Plastic Surgery *(ASAPS)* members, showed that plastic surgeons’ estimation of BDD in their cosmetic practice is only 2%. Thus, given the documented prevalence of BDD in cosmetic settings (Sweis et al., 2017; Anonymous, 1991; Ramos et al., 2019), and the current study findings, cosmetic treatment providers did indeed underestimate the prevalence of BDD.

**Table 4:** Experiences with body dysmorphic disorder patients. 3–2 ×Specialty *p* < .049 4–3 ×Specialty *p* < .007.

	**No. (%)**	**X2**	**df**	*p*
**1. The following is a list of Body Dysmorphic Disorder symptoms, please select the symptoms that you have most frequently encountered/ seen in patients of whom you suspected to have BDD (check all that apply)?**				
**A. Excessive concern with, or distress over, minor or nonexistent appearance flaws**	97 (62.6%)	5.089	3	.165
**B. Dissatisfaction with previous cosmetic surgery**	86 (55.5%)	2.904	3	.407
**C. Unusual or excessive requests for cosmetic surgery**	94 (60.6%)	7.722	3	.052
**D. References to others taking special note of the perceived appearance flaw**	28 (18.1%)	6.693	3	.082
**E. Belief that the procedure will transform patient’s life or solve all problems**	93 (60%)	3.919	3	.270
**F. Camouflaging (heavy makeup or clothes that hide body)**	36 (23.2%)	7.977	3	.046
**G. Difficulty in day-to-day functioning**	18 (11.6%)	8.135	3	.043
**H. Skin picking**	22 (14.3%)	7.284	3	.063
**2. Do you explore BDD or Disturbed Body Image during the initial interview?**^**3**^				
**A. Never**	14 (9%)	23.938	9	.006
**B. Sometimes**	85 (54.8%)			
**C. Often**	36 (23.2%)			
**D. Always**	20 (12.9%)			
**3. In patients with BDD, do you shift the topic from the technical aspects of the procedure to body image problems?**^**4**^				
**A. No**	14 (9%)	9.248	9	.415
**B. Sometimes**	82 (52.9%)			
**C. Most of the time**	34 (21.9%)			
**D. Always**	25 (16.1%)			
**4. In cases of BDD, do you share this knowledge with your patient?**				
**A. No**	47 (30.3%)			
**B. Sometimes**	76 (49%) 13.408 9 .145
**C. Most of the time**	20 (12.9%)			
**D. Always**	12 (7.7%)			
**5. What do you do when you recognize or suspect BDD in a patient?**				
**A. I don’t address this**	25 (16.1%)			
**B. Same approach to other patients**	28 (18.1%)			
**C. Share my impression on the patient’s appearance**	20 (12.9%)	29.686	21	.098
**D. I talk about the patient’s disturbed body image**	24 (15.5%)			
**E. First, I consult a psychologist about what to do**	23 (14.8%)			
**F. I refer the patient to a psychiatrist or a psychologist and decline the procedure**	13 (8.4%)			
**G. First, I refer the patient to a psychiatrist, and possibly carry out the requested procedure later**	17 (11%)			
**H. I carry out the procedure in parallel with psychological care**	5 (3.2%)			
**6. Have you been threatened by a patient with BDD?**				
**A. No, never**	130 (83.9%)			
**B. I have been physically threatened**	3 (1.9%)			
		10.475	9	.313
**C. I have been verbally threatened**	15 (9.7%)			
**D. I have been threatened with legal steps**	7 (4.5%)			

With regard to the differences between male and female patients in terms of BDD occurrence, the study by AlShahwan (2020), which was conducted in Saudi Arabia to estimate BDD prevalence found a higher prevalence of female (16.8%) versus male (5%) patients. Most of the physicians in the current study believe BDD prevalence to be equal between genders which is consistent with Sarwar study on *ASAPS* members (Sarwer, 2002).

Most participants were certain about encountering patients with BDD during last year, which is similar to what was reported in Bouman et al.’s study, where (62%) of participants encountered BDD patients (Bouman, Mulkens & Van der Lei, 2017). Szepietowski et al. (2008) study on polish dermatologists found that (64%) of participants encountered patients with BDD during their years of service. The current study has also shown that half of all the physicians who were completely familiar with the diagnostic criteria of BDD were certain they encountered BDD patients within the last year. Thus, it can be inferred that comprehensive knowledge of diagnostic criteria for BDD may lead to an increased number of correct BDD diagnoses, as well as less undiagnosed cases.

The physicians in the current study consider an encounter with BDD patients challenging, which is a common finding with Bouman, Mulkens & Van der Lei (2017) study. Two-thirds said they would not perform an unnecessary cosmetic procedure, and less than (10%) said they would. They also believe that BDD patients do not have realistic expectations on the impact of aesthetic procedures on their daily functioning, nor the results of the procedure itself. Participants in Szepietowski et al. (2008), and Bouman, Mulkens & Van der Lei (2017) agreed on the same opinion. Recent literature indeed reported that BDD patients would not be satisfied by the results post-procedure (Crerand et al., 2005), with (6%) of patients satisfied (Sarwer, 2002), and only (1%) free of preoccupation (Crerand et al., 2005). Furthermore, they tend to be more preoccupied with the previous defect or find a new defect to focus on (Crerand et al., 2005).

Approximately half of all participants agreed that BDD is a contraindication for cosmetic procedures. In comparison to other studies, only (30%) of *ASAPS* members considered BDD a contraindication for cosmetic surgery (Sarwer, 2002). The study by Sarwer et al. on dermatologic surgeons, found that two-thirds believe BDD a contraindication for surgery (Sarwer et al., 2015). Moreover, Bouman, Mulkens & Van der Lei (2017), found that almost (70%) of their sample perceives BDD a contraindication. While Cosmetic professionals agreed to varying extents that BDD is a contraindication for cosmetic surgery, a recent study by Felix et al. (2014) suggests that patients with mild to moderate degrees of BDD severity may benefit from cosmetic treatment.

As for physicians’ preference when dealing with a patient suspected of having BDD, most of the current participants would approach them the same way as any other patient or would not address the issue of BDD. In comparison to Bouman, Mulkens & Van der Lei (2017), the majority of the professionals would address and talk to the patient about BDD, and only a few would approach the patients with BDD the same as any other patient. In addition, only (37%) of the current participants would involve psychological care in the management of these patients. While the majority in Bouman, Mulkens & Van der Lei (2017), Sarwer et al. (2015), and Szepietowski et al. (2008), would involve a psychiatrist. These differences in preference between Saudi and other professionals may be due to cultural reasons. The conservative nature of the Saudi population may stigmatize a patient labeled with BDD, as such physicians would opt to treat these individuals similar to the non-BDD patients.

The most commonly observed symptoms of BDD by the current participants were excessive concern with minor appearance flaws, excessive requests for cosmetic surgery, and a belief that the procedure will solve all his problems. The results are expected given the reason that these patients are visiting a cosmetic physician. Generally, these results are consistent with Sarwar study (Sarwer, 2002).

Regarding the possible verbal and physical abuse that a professional might have faced, (83%) of our participants have not been threatened by a patient with BDD, while (9.2%) have been verbally threatened, and only (1%) have been physically threatened. This is partly consistent with Bouman, Mulkens & Van der Lei (2017), where (77%) of the participants have not been threatened by patients with BDD, and only (16.2%) have been threatened verbally (Anonymous, 1991). On the other hand in Sarwer’s study (40%) of their participants have been threatened by patients with BDD (Sarwer, 2002).

Limitations of this study include the subjective nature of self-assessment by the physicians regarding their familiarity with BDD and its manifestations. This is in addition to the low sample size and convenience sampling technique. The study also yielded little statistical significance concerning the specialty. Finally, the scope of the study was limited to the city of Riyadh and Jeddah and would benefit from including other cities within Saudi Arabia and possible other Middle Eastern countries.

In the future, the study may be augmented by developing a validated scale that accurately evaluates the physicians on their factual knowledge of BDD, and by developing multiple Arabic versions of screening tools to assess the severity of BDD symptoms as utilized in a study by Ramos et al. (2019). Finally, one of the long-term goals of our research team is to implement a validated BDD screening tool for cosmetic treatment providers to use in their practice.

## Conclusion

We have identified a discrepancy between the self-reported participant knowledge of diagnostic criteria and clinical presentation of BDD and their ability to accurately estimate the prevalence of BDD cases seen in practice. We also observed that most participants do not, or only minimally discuss BDD with patients they suspect of having BDD. As such, cosmetic treatment providers should aim to be more familiar with the clinical guidelines and diagnostic criteria of BDD to effectively manage these patients and spare them the monetary, physical and psychological toll of unnecessary procedures.

##  Supplemental Information

10.7717/peerj.8959/supp-1 Supplemental Information 1 BDD awareness SPSS form

10.7717/peerj.8959/supp-2 Supplemental Information 2 Saudi BDD questionnaire

## Additional Information and Declarations

### Data Availability

The following information was supplied regarding data availability: Raw data is available as a Supplemental File.

## References

[ref-1] Al Arfaj AM, Al Otaibi TM, Obeid AA, Alkhunaizi AA, Subhan YS, Al Arfaj A (2016). 1. Development validation and testing of an arabic version of the cosmetic procedure screening questionnaire COPS for body dysmorphic disorder. Kuwait Medical Journal.

[ref-2] Al Shuhayb ZS (2019). Prevalence of body dysmorphic disorder among Saudis seeking facial plastic surgery. Saudi Surgical Journal.

[ref-3] Alonazi H, Alharbi M, Alyousif L, Alialaswad W, Alharbi J, Almalki M, Alrashedee B (2017). Prevalence of body dysmorphic disorder in patients attending dermatology clinic in Saudi Arabia/Qassim region. Journal of Medical Science And Clinical Research.

[ref-4] AlShahwan MA (2020). Prevalence and characteristics of body dysmorphic disorder in Arab dermatology patients. Saudi Medical Journal.

[ref-5] American Psychiatric Association (1994). Diagnostic and statistical manual of mental disorders.

[ref-6] American Psychiatric Association (2013). Diagnostic and statistical manual of mental disorders.

[ref-7] (1991). Body dysmorphic disorder: the distress of imagined ugliness. American Journal of Psychiatry.

[ref-8] Bouman T, Mulkens S, Van der Lei B (2017). Cosmetic professionals’ awareness of body dysmorphic disorder. Plastic and Reconstructive Surgery.

[ref-9] Crerand CE, Phillips KA, Menard W, Fay C (2005). Nonpsychiatric medical treatment of body dysmorphic disorder. Psychosomatics.

[ref-10] Felix GA, De Brito MJ, Nahas FX, Tavares H, Cordás TA, Dini GM, Ferreira LM (2014). Patients with mild to moderate body dysmorphic disorder may benefit from rhinoplasty. Journal of Plastic, Reconstructive & Aesthetic Surgery.

[ref-11] Koran L, Abujaoude E, Large M, Serpe R (2008). The prevalence of body dysmorphic disorder in the United States adult population. CNS Spectrums.

[ref-12] Phillips KA (2005). Understanding and treating body dysmorphic disorder.

[ref-13] Ramos TD, De Brito MJA, Suzuki VY, Sabino Neto M, Ferreira LM (2019). High prevalence of body dysmorphic disorder and moderate to severe appearance-related obsessive-compulsive symptoms among rhinoplasty candidates. Aesthetic Plastic Surgery.

[ref-14] Sarwer DB (2002). Awareness and identification of body dysmorphic disorder by aesthetic surgeons: results of a survey of the American Society for Aesthetic Plastic Surgery members. Aesthetic Surgery Journal.

[ref-15] Sarwer DB, Spitzer JC, Sobanko JF, Beer KR (2015). Identification and management of mental health issues by dermatologic surgeons: a survey of American Society for Dermatologic Surgery members. Dermatologic Surgery.

[ref-16] Shaffi Ahamed S, Enani J, Alfaraidi L, Sannari L, Algain R, Alsawah Z, Al Hazmi A (2016). Prevalence of body dysmorphic disorder and its association with body features in female medical students. Iranian Journal of Psychiatry and Behavioral Sciences.

[ref-17] Sweis I, Spitz J, Barry D, Cohen M (2017). A review of body dysmorphic disorder in aesthetic surgery patients and the legal implications. Aesthetic Plastic Surgery.

[ref-18] Szepietowski JC, Salomon J, Pacan P, Hrehorów E, Zalewska A (2008). Body dysmorphic disorder and dermatologists. Journal of the European Academy of Dermatology and Venereology.

