# Supplementary material for: Awareness and experiences of cosmetic treatment providers with body dysmorphic disorder in Saudi Arabia
Source: PeerJ. 2020 Apr 21;8:e8959. doi: 10.7717/peerj.8959 (PMC7182027; doi:10.7717/peerj.8959)
Supplement: Supplemental Information 2 [file peerj-08-8959-s002.docx]

***Section 1: Demographics and characteristics of respondents***

**Gender**

- Female
- Male

**Age (in years)** _______________________________________________

**Specialty**

- Plastic surgeon
- Dermatologist
- Otorhinolaryngologist

**Nationality**

- Saudi
- Non-Saudi (please mention the nationality)
  - ___________________________________________________________________

**Does your practice involve the provision of *cosmetic treatments*?**

- Yes
- No

**Years of experience**

- Less than 5
- 5 to 10
- 10 to 15
- 15 to 20
- more than 20

**No. of new patients per year**

- <200
- >200–350
- >350–500
- >500

***Section 2: Awareness of Body Dysmorphic Disorder***

***Body Dysmorphic Disorder (BDD) is a formal psychiatric diagnosis defined as an excessive preoccupation with an imagined or slight defect in appearance which leads to significant distress or impairment in social, occupational or other areas of functioning. Please use this description as you answer the following questions.***

1. **Are you familiar with the diagnostic criteria for BDD?**
   1. I’m seeing these criteria now for the first time
   2. I’ve heard of these
   3. I’m slightly familiar with these
   4. I have been familiar with these for a long time
2. **Are you familiar with the clinical picture of BDD?**a. I’m not familiar with this
   b. I’m partly familiar with this
   c. I’m reasonably familiar with this
   d. I’m totally familiar with this
3. **How did you Acquire knowledge on Body Dysmorphic Disorder?**
4. General professional Literature
5. Specific Literature on BDD
6. Conferences or Lecture
7. Colleagues
8. Web sites
9. **What is your estimate of the percentage of all patients seen for an initial cosmetic consultation in your practice who you believe to suffer from BDD?**
10. Approximately 1% - 5%
11. Approximately 5%-10%
12. Approximately 10% - 15%
13. Approximately 15 % - 20%
14. I have no idea
15. **What percentage of *MALE* patients seen for an initial cosmetic consultation do you estimate suffer from BDD?**
    1. Approximately 5% - 10%
    2. Approximately 10% - 20
    3. Approximately 20 %– 30%
    4. I have no idea
16. **What percentage of FEMALE patients seen for an initial cosmetic consultation do you estimate suffer from BDD?**
    1. Approximately 5% - 10%
    2. Approximately 10% - 20
    3. Approximately 20 %– 30%
    4. I have no idea
17. **Did you encounter Patients with BDD over the past year?**
    1. Yes, I have probably encounter them
    2. Yes, I have certainly encounter them
    3. No, I did not encounter any
18. **How many patients with BDD did you see last year?**
    1. None
    2. 1 -5 patients
    3. 5 – 10
    4. More than 10 patients

***Section 3: Attitudes toward BDD and its impact on cosmetic practice***

**Please note that: 1 = don’t agree at all; 3= neutral; 5 = totally agree.**

| 1. **I usually get a gut feeling that something is wrong when seeing patients with BDD** | 1 | 2 | 3 | 4 | 5 |
| --- | --- | --- | --- | --- | --- |
| 1. **I find it challenging to deal with patients with BDD** | 1 | 2 | 3 | 4 | 5 |
| 1. **I find BDD a contraindication for an aesthetic procedure** | 1 | 2 | 3 | 4 | 5 |
| 1. **Patients with BDD have realistic expectations on the impact of the aesthetic procedure on their daily functioning** | 1 | 2 | 3 | 4 | 5 |
| 1. **Patients with BDD have realistic expectations on the physical results of the aesthetic procedure** | 1 | 2 | 3 | 4 | 5 |
| 1. **Patients with BDD benefit equally from aesthetic procedures as other patients** | 1 | 2 | 3 | 4 | 5 |
| 1. **If a patient wants an aesthetic procedure, I will always carry this out** | 1 | 2 | 3 | 4 | 5 |
| 1. **If I think an aesthetic procedure to be unnecessary, I’ll tell the patient** | 1 | 2 | 3 | 4 | 5 |
| 1. **Even if I find an aesthetic procedure unnecessary, I’ll still carry this out** | 1 | 2 | 3 | 4 | 5 |
| 1. **If I find an aesthetic procedure unnecessary, I refer the patient to a colleague** | 1 | 2 | 3 | 4 | 5 |
| 1. **Aesthetic procedures are a luxury article, but also patient care** | 1 | 2 | 3 | 4 | 5 |
| 1. **Aesthetic procedures are basically a kind of “psychotherapy/psychosurgery** | 1 | 2 | 3 | 4 | 5 |

***Section 4: Experiences with Body Dysmorphic Disorder Patients***

***Assuming that you frequently see clients with typical clinical picture of BDD, we would like to know how you have been dealing and managing these clients.***

1. **The following is a list of Body Dysmorphic Disorder symptoms, please select the symptoms that you have most frequently encountered/ seen in patients of whom you suspected to have BDD *(check all that applies)*?**
   1. Excessive concern with, or distress over, minor or nonexistent appearance flaws
   2. Dissatisfaction with previous cosmetic surgery
   3. Unusual or excessive requests for cosmetic surgery
   4. References to others taking special note of the perceived appearance flaw
   5. Belief that the procedure will transform patient’s life or solve all problems
   6. Camouflaging (heavy makeup or clothes that hide body)
   7. Difficulty in day-to-day functioning
   8. Skin picking
2. **Do you explore BDD or Disturbed body image during the initial interview?**
   1. Never
   2. Sometimes
   3. Often
   4. Always
3. **In patients with BDD, do you shift the topic from the technical aspects of the procedure
   to body image problems**

a. No. Body image is never part of the intake interview
 b. Sometimes
 c. Most of the time
 d. Always, because body image is a standard topic during the intake interview

1. **In case of BDD, or disturbed Body image, do you share this with your patient?**
   1. No, I keep that to myself
   2. Sometimes
   3. Most of the time
   4. Yes, always
2. **What do you do when you recognize or suspect BDD in a patient?**
   a. I don’t address this
   b. I approach such a patient no different from other patients
   c. I share my impression on this patient’s appearance

d. I talk about the patient’s disturbed body image
e. First, I consult a psychologist about what to do

f. I refer the patient to a psychiatrist or a psychologist and decline the procedure
g. First, I refer the patient to a psychiatrist or a psychologist, and possibly carry out the requested procedure later
h. I carry out the procedure in parallel with psychological care

1. **Have you ever been threatened by a patient with BDD?**a. No, this has never happened to me
   b. I have been physically threatened

c. I have been verbally threatened
d. I have been threatened with legal steps

1. **Would you be interested in providing information to prospective patients about BDD (If answered Yes, please answer question 8)?**
   1. Yes
   2. No
2. **How would you like to distribute this information?**
   1. Direct patients to a website about BDD
   2. Provide patients with a brochure on BDD
   3. Recommend a book to patients about BDD
   4. Others (Please write the method that you prefer to distribute the information)
      1. ___________________________________________________________________
